# Essential oil of *Lippia alba* and its main constituent citral block the excitability of rat sciatic nerves

**DOI:** 10.1590/1414-431X20154710

**Published:** 2015-06-30

**Authors:** D.G. Sousa, S.D.G. Sousa, R.E.R. Silva, K.S. Silva-Alves, F.W. Ferreira-da-Silva, M.R. Kerntopf, I.R.A. Menezes, J.H. Leal-Cardoso, R. Barbosa

**Affiliations:** 1 Laboratório de Fisiofarmacologia das Células Excitáveis, Universidade Regional do Cariri, Crato, CE, Brasil; 2 Laboratório de Farmacologia e Química Medicinal, Universidade Regional do Cariri, Crato, CE, Brasil; 3 Programa de Mestrado em Bioprospecção Molecular, Universidade Regional do Cariri, Crato, CE, Brasil; 4 Laboratório de Eletrofisiologia, Instituto Superior de Ciências Biomédicas, Universidade Estadual do Ceará, Fortaleza, CE, Brasil

**Keywords:** Citral, Nerve excitability, *Lippia alba*, Sciatic nerve, Essential oil, Compound action potential

## Abstract

*Lippia alba* is empirically used for infusions, teas, macerates, and hydroalcoholic extracts because of its antispasmodic, analgesic, sedative, and anxiolytic effects. Citral is a mixture of trans-geranial and cis-neral and is the main constituent of *L. alba* essential oil and possesses analgesic, anxiolytic, anticonvulsant, and sedative effects. The present study evaluated the effects of the essential oil of *L. alba* (EOLa) and citral on compound action potentials (CAPs) in Wistar rat sciatic nerves. Both drugs inhibited CAP in a concentration-dependent manner. The calculated half-maximal inhibitory concentrations (IC_50_) of peak-to-peak amplitude were 53.2 µg/mL and 35.00 µg/mL (or 230 µM) for EOLa and citral, respectively. Peak-to-peak amplitude of the CAP was significantly reduced by 30 µg/mL EOLa and 10 µg/mL citral. EOLa and citral (at 60 and 30 µg/mL, values close to their respective IC_50_ for CAP blockade) significantly increased chronaxy and rheobase. The conduction velocity of the first and second CAP components was statistically reduced to ∼86% of control with 10 µg/mL EOLa and ∼90% of control with 3 µg/mL citral. This study showed that EOLa inhibited nerve excitability and this effect can be explained by the presence of citral in its composition. Both EOLa and citral showed inhibitory actions at lower concentrations compared with other essential oils and constituents with local anesthetic activity. In conclusion, these data demonstrate that EOLa and citral are promising agents in the development of new drugs with local anesthetic activity.

## Introduction

Essential oils are natural volatile substances with a strong odor among many characteristics, which originate from the secondary metabolism of aromatic plants, and are extracted from several vegetal organs. Essential oils have antiseptic and medicinal properties, and are used as analgesic, sedative, antimicrobial, anti-inflammatory, antispasmodics, and local anesthetics. (1).

*Lippia alba* is popularly called erva-cidreira (in Portuguese) and belongs to the Verbenaceae family. It is a vegetal of shrubby habit and is found in almost all Brazilian territories. It is empirically used for teas, alcoholic extracts, macerates, compress preparations, baths, antipyretics, anti-inflammatory agents, to aid the stomach, and as an analgesic and a sedative. *L. alba* also possesses low toxicity and its efficacy is attributed to its main constituents (2).

Citral is a major constituent found in the essential oil of *L. alba* (EOLa) and is a mix of two isomers that are structurally different but have the same molecular formula: trans-geranial and cis-neral (3). Several studies have reported the pharmacological actions of citral, which include spasmodic, anti-inflammatory (4), sedative (5), and antinociceptive (6) effects. Citral is reported to have low toxicity, because doses lower than 1068 g/kg do not show toxicity in rats (7). Citral also targets transient receptor potential (TRP) channels in sensory neurons producing a long duration inhibition of TRPV1-3 and TRPM8 and transitory inhibition of TRPV4 and TRPA1, suggesting that it has greater efficacy than capsaicin for allodynia, itching, and certain types of pain that affect sensorial nerves and corporal surfaces (8). These studies highlight the need for research to focus on the actions of citral on nerve fibers.

The characteristics of low toxicity and effects on the nervous system indicate that EOLa and its main constituent citral might have effects on the compound action potential (CAP) of rat sciatic nerve. Several essential oils and their main constituents promoted alterations of peripheral nerve activity ([Bibr B09] [Bibr B10] [Bibr B11] 912). No previous studies have characterized the effects of EOLa and citral on peripheral nerve function, therefore, the present study investigated the actions of EOLa and citral on the CAPs recorded in the sciatic nerve of Wistar rats.

## Material and Methods

### Vegetal material and chemical analysis

The essential EOLa was purchased from Prof. Dr. Sergio Horta. L. alba voucher was deposited in the Prisco Bezerra Herbarium (Federal University of Ceará) with the following number identification #EAC-08474. Essential oil samples were analyzed by gas-liquid chromatography coupled to mass spectrometry at Parque de Desenvolvimento Tecnológico. The main compounds detected were citral 75% [geranial (41.81%), neral (34.11%)], 1-limoneno (9.85%), carvone (8.92%), gamma-terpinene (2.05%), benzene, and 1-methyl-3-(1-methylethyl) (1.02%).

### Solution and drugs

For nerve dissection and extracellular recordings, modified Locke's solution was used with the following composition: 140 mM NaCl, 5.6 mM KCl, 2.2 mM CaCl_2_, 1.2 mM MgCl_2_, 10 mM glucose, 10 mM Tris-hydroxymethyl aminomethane (TRIS), pH adjusted to 7.4 with HCl or NaOH. To prepare the drugs at the desired concentrations, EOLa and citral were first dissolved in dimethylsulfoxide (DMSO) and then diluted in Locke's solution to obtain working solutions (3, 10, 30, 60, 100, 300, and 1000 µg/mL). Locke’s solution with maximal DMSO employed (0.2% v/v) was used as the control. All salts and DMSO were of analytical grade and purchased from Sigma-Aldrich (USA).

### Animals and nerve dissection

Sciatic nerves were dissected from Wistar rats (*Rattus norvegicus*, body weight 200-250 g) of both sexes. The animals were euthanized in a CO_2_ chamber and subsequently both sciatic nerves were removed. The sciatic nerves were stored in modified Locke's solution at room temperature until electrophysiological recordings.

### Extracellular recording

Sciatic nerves were mounted in a moist chamber and were stimulated continuously at a frequency of 0.2 Hz with an electric pulse of 100-200 ms duration, amplitude 20-40 V, delivered by a stimulus isolation unit (Model SIU4678, Grass Instruments, USA), connected to a stimulator (Model S48, Grass Instruments). The CAP was evoked at one end by platinum wires (stimulating electrodes). The evoked signal was recorded at the other extremity of the nerve with a recording electrode placed 40-50 mm from the stimulating electrodes and monitored using an oscilloscope (Model 547, Tektronix, Inc., USA). Data was continuously stored in a personal computer by an acquisition software/hardware system (pClamp 9/Digidata 1332A, Molecular Devices, USA) for further analysis. To administer EOLa and citral and maintain chamber humidity, a segment of the sciatic nerve (15-20 mm) was suspended between the stimuli and recording electrodes and immersed in Locke's solution. EOLa and citral were applied to the sciatic nerve only when stable peak-to-peak amplitude (PPA) of CAP was achieved for at least 30 min (varying by less than 5% in amplitude), and with an exposure time of 180 min. This period was followed by a recovery period of 180 min. The electrophysiological parameters measured by extracellular recording were rheobase, chronaxy, PPA, and conduction velocity of CAP components. Rheobase was defined as the threshold stimulus voltage for an active response with a long duration pulse (1000 μs), and chronaxy as the threshold duration for an active response with a stimulus twice rheobase.

### Statistical analysis

Data are reported as means±SE. The letter “n” indicates the number of experiments. We used a paired *t*-test to compare two samples, and for multiple comparison testing we used a one-way ANOVA followed by a *post hoc* test (Dunnett's). Two means were considered statistically different when P<0.05. The IC_50_ of EOLa and citral were calculated from the concentration response curves, where the experimental points were fitted by a Hill equation.

## Results

The electrophysiological parameters PPA, conduction velocity of CAP first and second components, rheobase, and chronaxy were analyzed after nerve stabilization and the control values were 7.1±0.27 mV, 85.4±2.34 m/s, 30.8±1.11 m/s, 3.4±0.22 V, and 51.5±2.34 µs (n=87), respectively.

The obtained CAP register showed the electrical activity of axons in a nerve bundle. We identified the presence of two CAP components and based on calculated conduction velocities of each wave and by comparing them to conduction velocities of mammalian fibers, we distinguished the fiber types in each component. The first CAP component had a mean conduction velocity of 85.4±2.34 m/s and this range of velocity is characteristic of Aα motor fibers. For the second CAP component, the mean conduction velocity was 30.8±1.11 m/s and this velocity comprises Aβ myelinated sensory fibers and Aγ myelinated motor fibers. Our results showed that EOLa and citral fully blocked the CAP waves. All EOLa and citral concentrations used preferentially affected the second CAP component, indicating a major preference of EOLa and citral for small diameter myelinated fibers.

**Figure 1:**
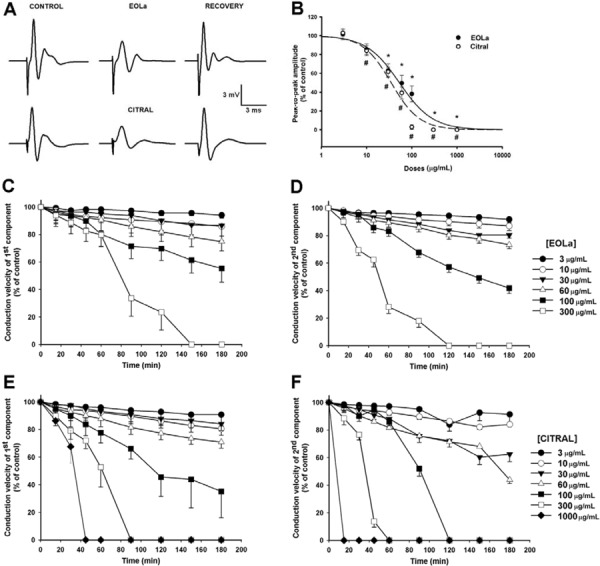
Representative traces and data of rat sciatic nerve compound action potentials (CAP) in control, essential oil of *Lippia alba* (EOLa) and citral groups. *Panel A*: left traces indicate CAP in control group; center traces indicate nerve exposed to 60 µg/mL EOLa and 60 µg/mL citral; and right traces indicate the recovery period. Concentration-dependent curves for EOLa and citral on peak-to-peak amplitude of CAP are shown in *panel B*. *Panels C* and *D* show the time course of conduction velocities of the first and second CAP components upon exposure to EOLa. *Panels E* and *F* show the effects of citral on conduction velocity time course. P<0.05, *EOLa and ^#^citral compared to control (ANOVA or paired *t*-test).

Figure 1 shows the two CAP components of sciatic nerves at the end of the stabilization period (left traces). When applied to the sciatic nerve, EOLa and citral (60 and 30 µg/mL, respectively) progressively blocked the amplitude of CAP waves during 180 min of drug exposure (Figure 1A and B) reaching 50% of PPA (center traces). Removal and replacement of EOLa and citral solutions with Locke's solution promoted the recovery of CAP components similar to the control traces (right traces).

The threshold concentrations (concentrations that produced a significant reduction in the PPA of the CAP) for EOLa (n=7) and for citral (n=6) were 30 µg/mL (62.8±7.10% of control) and 10 µg/mL (84.1±5.38% of control), respectively. Complete block of CAP by EOLa occurred with 300 µg/mL. At the end of 180 min exposure to citral 100 µg/mL, the PPA reduction value was 2.9±2.54% (n=4) and 300 µg/mL citral completely abolished CAP components. The depressor effects of citral on CAP components of the sciatic nerve were more potent than for EOLa because 300 µg/mL citral completely blocked CAP within 90 min, whereas for EOLa complete blockade was achieved at 180 min of drug exposure.

Citral, the major constituent of EOLa, displays similar effects to the essential oil and is likely responsible for the action of essential oil on the excitability of sciatic nerves. This is because EOLa-induced inhibition on PPA was significant at a concentration of 30 µg/mL, and the sample of EOLa used is approximately 75% citral (22 µg/mL) and citral depresses excitability at concentrations ≥10 µg/mL. Thus, citral may be considered the primary component responsible for the action of EOLa.

The conduction velocity of CAP components was reduced in a concentration-dependent manner for both EOLa and citral. EOLa significantly reduced this parameter (P<0.05, paired *t*-test) at a concentration range of 10-100 µg/mL (Figure 1D) and 10 µg/mL EOLa reduced the first and second component conduction velocities to 85.8±3.87 and 86.9±2.68% (n=5) of the control values, respectively. All citral concentrations used (3-60 µg/mL) induced a statistically significant reduction of both CAP components (P<0.05, paired *t*-test). For citral at 100 and 300 µg/mL and EOLa at 300 µg/mL, the CAP amplitudes were blocked in such a way that we were not able to measure the conduction velocities of both components.

The nerve excitability was assessed by the quantification of rheobase and chronaxy parameters. Thus, adjusting the concentrations used and PPA data with a logistic equation allowed us to calculate the IC_50_, in which the EOLa and citral values were 53.1±15.81 µg/mL (37.38-69 µg/mL range) and 35.0±13.45 µg/mL (21.6-48.5 µg/mL range), respectively (Figure 1B).

**Figure 2:**
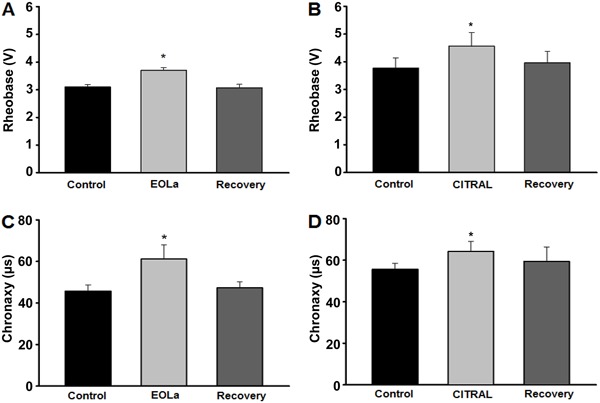
Alterations in rheobase (*A*, *B*) and chronaxy (*C*, *D*) after exposure to essential oil of *Lippia alba* (EOLa) and citral. *P<0.05, compared to control (ANOVA or paired *t*-test)**.**

In view of all the concentrations used, we chose 60 µg/mL EOLa and 30 µg/mL citral (concentration values near to the IC_50_ for both drugs) to study their effects on nerve excitability (Figure 2). At the end of 180 min exposure to EOLa and citral, rheobase was increased to 3.7±0.09 (n=7) and 4.6±0.54 V (n=9), respectively. Chronaxy was significantly altered to 61.1±6.80 µs (n=7) and 65.2±5.26 µs (n=9) for 60 µg/mL EOLa and 30 µg/mL citral, respectively compared to controls (both, P<0.05, paired *t*-test). Thus, the presence of EOLa and citral reduced nerve excitability due to the increased values of rheobase and chronaxy, indicating a more potent stimulus with greater duration was required to generate an action potential (Figure 2).

## Discussion

This study showed that EOLa and its main constituent citral inhibited the excitability and conductibility of all myelinated fiber types that contribute to the CAP components of sciatic nerve in a concentration-dependent manner. The effects of both drugs were reversible after washout and showed greater potency in fibers with low conduction velocities.

It was observed that citral was responsible for the depressor effect of nerve excitability. This observation is in contrast with other essential oils where the major constituent was not responsible for excitability reduction, such as *Croton nepetaefolius* and its main constituent 1,8-cineole. Lima-Accioly et al. (10) showed that the essential oil *Croton nepetaefolius* significantly inhibited PPA of CAP at a concentration below 500 µg/mL, and this effect was seen only at a concentration of 614 µg/mL when using 1,8-cineole alone.

The conduction velocities of the two PAC components were altered by EOLa at a lower concentration than for other essential oils, such as *Croton nepetaefolius* and *Alpinia zerumbet,* which exhibited significant effects in the hundred µg/mL range (9,10). In contrast, citral had a depressor effect of conduction velocity at a dose of 3 µg/mL (equivalent concentration to 20 µM), which is lower than that for eugenol (60 µM), terpineol (300 µM) and estragole (2,000 µM) ([Bibr B09] [Bibr B10] 911).

Citral affected the conduction velocity of both CAP components, but myelinated Aβ sensory and Aγ motor fibers, which contribute to the second CAP component, were more sensitive to citral than fibers of the first CAP component. This behavior is in accordance with other terpenoids such as carvacrol and linalool and local anesthetics, such as lidocaine and bupivacaine ([Bibr B12] [Bibr B13] [Bibr B14] 1215).

The changes in excitability observed in the present study suggested that EOLa and citral possess local anesthetic activity. This is in agreement with previous studies showing that citral and *L. alba* possess antinociceptive activity (11).

Several constituents from medicinal plants such as estragole (16,17) and 1,8-cineole (18), act to reduce nerve excitability although their mechanism of action is only partially elucidated.

Little is known about the mechanism of action of EOLa and citral on the reduction of nerve excitability. Therefore, further studies are required. However, EOLa and citral showed similar effects to other constituents in the same class of terpenes, such as carvacrol and linalool (12,13,19,20). It was already demonstrated that linalool and carvacrol block neuronal excitability by a direct action on voltage dependent Na+ channels, and it is reasonable to suggest a similar mechanism of action for EOLa and citral in reducing excitability.

In conclusion, we demonstrated that EOLa and citral depressed nerve excitability in a concentration-dependent manner. All electrophysiological parameters analyzed showed that citral was more potent than EOLa. The modifications on excitability induced by EOLa and citral were observed at lower concentrations than those required by other essential oils. In summary, EOLa and citral demonstrated a local anesthetic activity. The data presented here might contribute to the development of new drugs with anesthetic actions. Furthermore, *L. alba* and its main constituent citral could be potential candidates for anesthetics in clinical trials.
